# CCP11 Group Meeting—Towards the Functional Analysis of Microarrays

**DOI:** 10.1002/cfg.201

**Published:** 2002-10

**Authors:** Clare Sansom

**Affiliations:** School of Crystallography Birkbeck College University of London Malet Street London WC1E 7HX United Kingdom

## Abstract

The CCP11 project [2] aims to foster bioinformatics in the UK through conferences, workshops and the provision of Web resources. In March 2002, CCP11 held a meeting in Manchester, UK, on the functional analysis of microarrays. This was part of Manchester BioinformaticsWeek—three consecutive short bioinformatics meetings held in the attractive setting of the Chancellor's Conference Centre at the University of Manchester. The other meetings in the series were a workshop on ontologies and the 12th Annual MASAMB (Mathematical and Statistical Aspects of Molecular Biology) Conference. Many delegates were able to attend more than one meeting, which led to a useful cross-fertilization of ideas across the bioinformatics community. The CCP11 meeting shared with MASAMB a strong emphasis on the statistical analysis and interpretation of data—most often image intensity data.


**Comparative and Functional Genomics**
*Comp Funct Genom* 2002; **3**: 451–454.
Published online 29 August 2002 in Wiley InterScience (www.interscience.wiley.com). **DOI:** 10.1002/cfg.201

**Feature**

### Meeting Review: CCP11 Group Meeting — Towards the Functional Analysis of Microarrays

Chancellor's Conference Centre, University of Manchester, UK, 27–28 March 2002

Clare Sansom*
*School of Crystallography, Birkbeck College, University of London, Malet Street, London WC1E 7HX, UK*

**Correspondence to:*
*Clare Sansom, School of Crystallography, Birkbeck College, University of London, Malet Street, London WC1E 7HX, UK.*
*E-mail:*
*c.sansom@mail.cryst.bbk.ac.uk*

Received: 12 June 2002
Accepted: 17 June 2002

The CCP11 project [2] aims to foster bioinformatics in the UK through conferences, workshops and the provision of Web resources. In March 2002, CCP11 held a meeting in Manchester, UK, on the functional analysis of microarrays. This was part of Manchester Bioinformatics Week — three consecutive short bioinformatics meetings held in the attractive setting of the Chancellor's Conference Centre at the University of Manchester. The other meetings in the series were a workshop on ontologies and the 12th Annual MASAMB (Mathematical and Statistical Aspects of Molecular Biology) Conference. Many delegates were able to attend more than one meeting, which led to a useful cross-fertilization of ideas across the bioinformatics community. The CCP11 meeting shared with MASAMB a strong emphasis on the statistical analysis and interpretation of data — most often image intensity data.

### Introduction

**Tom Freeman (Human Genome Mapping Project Resource Centre, Hinxton, Cambridge, UK)** set the scene by describing the HGMP-RC's comprehensive microarray service covering array design, distribution and data release [6]. This service is funded by the Medical Research Council with the aim of centralizing microarray production and training in the UK. The two main technology platforms are both supported. Scientists working with spotted DNA arrays are provided with a full service, ranging from array design and fabrication through to data analysis, whilst those working with the commercial Affymetrix Gene Chip system are helped with protocol design and analysis. Freeman described a number of so-called 'golden rules' for successful microarray analysis. He advised researchers to isolate the process under study, be aware of the limitations of the techniques, use both biological and experimental replicates, and verify and follow-up their results.

Other speakers focused on the challenges of storing the many gigabytes of data produced by microarrays. This topic is sometimes neglected, as **Zlatko Trajanowski (Graz University of Technology, Austria)** pointed out: 'Many labs spend a

Copyright © 2002 John Wiley & Sons, Ltd.

lot of money on microarray production facilities but have just one PC for downstream processing'. His group is developing the Genome Information Management System (GIMS) to manage up to a terabyte of genomic, expression and related data at once. GIMS includes a relational database to hold the expression data, links to other local databases, and tools for storing, retrieving and analysing this data. The analysis software, which is mainly developed in Java, is free of charge [5]. His group is using GIMS for an analysis of lipid-associated disorders in the mouse. 'Snapshots' showing the expression of the approximately 32 000 mouse genes in different cell types under different conditions are stored in the database. This can be interrogated to answer questions like, 'Which genes are expressed in white adipose tissue in mice fed a high-fat diet?' and 'Which genes are overexpressed in any tissues in hyperlipidemia?'. Including data from gene knockout as well as wild-type mice has expanded the mouse database to over 30 million points of image data alone.

### Statistical methods and analysis

**Chris Glaseby (Biomathematics and Statistics Scotland, Edinburgh, UK)** addressed the first stage of the microarray analysis process: obtaining accurate and reproducible expression values from spot intensities. This process is complicated by 'noise' and irregularities in spot appearance and background intensity. He described the use of several successive filters to increase the signal : noise ratio, and compared $k$-means clustering to probabilistic methods in determining the ratio between the intensities of the two channels. He emphasized that it may be necessary to optimize parameters, and even choose analysis methods, independently for each application.

**David Hoyle (University of Manchester, UK)**, who described himself as a 'refugee physicist', reviewed the general characteristics of the intensity distribution in microarray datasets. Almost regardless of the source of the data, microarray intensity distributions tend to be asymptotic, with most genes expressed at low levels and a long 'tail' of a few highly expressed genes. Plotting the logarithm of the intensity data, however, will give a symmetric and approximately normal distribution. The variance of this distribution tends to be fairly constant for any species, but to increase with genome size. Applying the Central Limit Theorem and a generative model for mRNA abundance [1] to this problem suggested that the increase in variance was due to the increasing complexity of transcriptional control in species with larger genomes — most likely, to the increase in the average number of regulatory elements of each gene [7].

**Wolfgang Huber (DKFZ, Heidelberg, Germany)** described a statistical model for gene expression data, comprising data calibration and the quantification of differential expression and measurement error. The variance of intensity data increases as the mean intensity decreases, making estimation of differential expression difficult. He derived a transformation, $h(x) = arsinh(a + bx)$, in which the variance of $h(x)$ is independent of $x$. The parameters $a$ and $b$ will be constant for each channel of each experiment, and changes in expression can be estimated more accurately from differences in $h(x)$ than they can from the widely used log-differences.

Other speakers considered techniques for clustering genes based on their expression patterns. **Steve Swift (Brunel University, UK)** pointed out that, although many clustering methods are currently used, few groups have attempted to cross-compare the various methods or to check their consistency. He presented a comparison of five common methods: hierarchical clustering; $k$-means; self-organizing maps; hill climbing; and simulated annealing, and described algorithms to generate consensus clusters from all these methods. Testing these with both synthetic data and the herpes virus dataset used by Jenner and co-workers at University College, London (see below) he found the consensus to be more accurate than any single method. The consensus approach has been used successfully in other bioinformatics applications, such as the Jpred program for predicting the secondary structure of proteins [3].

Statistical techniques previously applied to disciplines ranging from economics to ecology can be applied to the interpretation of microarray intensity data. **Aedin Culhane (University of Cork, Ireland)** described the use of a technique that is used in ecology to classify cell lines based on their expression profiles. This technique, known as 'between-group Eigen analysis', is most useful in situations where there are more variables than samples. It involves combining variables to form

Copyright © 2002 John Wiley & Sons, Ltd.

discriminators that minimize the distance between group members while maximizing that between groups. Culhane and her co-workers were able to classify 33 out of 34 cell lines from the Golub leukaemia dataset [4] as acute myeloid leukaemia or acute lymphoblastic leukaemia, based only on their expression profiles.

Effective discrimination is particularly important in one of the best developed clinical uses of microarrays: differential diagnosis. **Richard Jenner** described his work in Paul Kellam's group at the **Wohl Virion Centre, University College London, UK,** using gene expression to distinguish between different types of B cell lymphoma. Lymphomas have been divided into over 20 different types, each with a different histology, treatment and prognosis: some are infected with characteristic herpes viruses. The London group created a microarray containing 5600 known cancer genes, together with genes from two herpes viruses, Kaposi's sarcoma-associated herpes virus and the Epstein–Barr virus. Using this, they were able to differentiate effectively between cell lines from a primary effusion lymphoma (infected with KSHV and EBV) and a previously misdiagnosed Burkitt's lymphoma (infected with EBV). Jenner also found that primary effusion leukaemia cells overexpressed the vitamin D receptor. He therefore predicted, and later proved *in vitro*, that this cell line would be sensitive to vitamin D analogues.

### Bacterial gene expression

About one-third of the world's population are infected with *Mycobacterium tuberculosis*. Although in the majority of cases the bacteria lie dormant in macrophages, this forms a vast reservoir of infection from which millions of overt tuberculosis cases arise each year. **Philip Butcher (St George's Hospital Medical School, London, UK)** presented the use of whole-genome *M. tuberculosis* microarrays to plot changes in gene expression during the bacterium's growth cycle. He has identified genes, including enzymes involved in the tricarboxylic acid cycle, that are specifically upregulated during the stationary phase. This is thought likely to be a good model for the bacterium in its latent form. **Graham Stewart (Imperial College, London, UK)** described a study of the response of *M. tuberculosis* to heat shock and other stresses, some of which can be taken as models for the conditions inside macrophages. He identified a number of novel heat shock proteins and two regulatory proteins that control their expression [8]. The most 'heat-shockable' tuberculosis gene of all was found to be an $\alpha$-crystallin that is related to a major antigen induced by anaerobic stress. Both these studies of *M. tuberculosis* gene expression provide insights into the biology of the organism in its dormant state, which could eventually lead to novel therapies for dormant tuberculosis.

**Colin Smith (UMIST, Manchester, UK)** presented an analysis of gene expression in the model streptomycete, *Streptomyces coelicolor*, which is a major producer of clinical and agricultural antibiotics. This bacterium has a large (8.7 Mb) genome, which contains some genes rarely found in prokaryotes. Smith and co-workers have been monitoring gene expression over time to identify those expressed during critical periods, such as commitment to differentiation and antibiotic production. The UMIST group is a major producer of *Streptomyces* microarrays, and these are available free of charge to the UK academic community from the UMIST microarray website [9]: the latest version, available since February 2002, includes probes for about 6800 genes.

### Modelling genetic networks

**David Wild (Keck Graduate Institute, California, USA)** described his use of linear dynamic systems to 'reverse-engineer' genetic networks from expression data. These are simple techniques for working out probable causes from given observables; in probability theory, they are a subclass of dynamic Bayesian networks. He illustrated the principle with a simple example, albeit one generally more applicable to California than to Manchester: 'Is the grass wet because it has been raining, or because the sprinkler has been on?' In microarray bioinformatics, the observables are the patterns of genes that are over- and underexpressed in different conditions: the causes to be inferred, networks of gene interactions. He used the model to infer a 'testable hypothesis' listing genes that influence each other during T cell activation and the generation of an immune response.

Copyright © 2002 John Wiley & Sons, Ltd.

**Johan Rung (EBI, Hinxton, UK)** presented a method for deducing gene dependency networks from microarray data. He compared the expression levels of all genes in each of 248 single gene deletion mutants of yeast, and identified all cases where the deletion of one gene (the source gene) caused a change in the expression of another (the target). These can be represented as graphs, with nodes representing genes and arrows joining those linked in this way. Genes in the same metabolic pathway were often located close together in the network. Genes that were the source for large number of interactions tended to be involved in regulation, and those that were the target for large numbers were involved in metabolism.

### Transcriptome analysis of single cells

Gene expression in some tissues can alter from one cell to the next. **Georgy Koentges (Wolfson Institute of Biomedical Research, University College London, UK)** presented an innovative technique that can be used to select single cells for transcriptome analysis. This technique is known as laser capture microscopy. An infrared laser beam with a diameter of 0.5–0.7 μm (similar to that of a single cell) is used to melt a polymer which wraps round the selected cell. The cell can be picked up with its polymer layer and its cDNA complement generated for analysis. Koentges has applied it to olfactory systems in embryonic mouse brain. Each receptor cell in these systems contains only one type of olfactory receptor, so it is easy to prove that single cells are being extracted. He has shown that it is possible to 'watch the genome landscape at work', even in such a tiny system.

### Conclusion

**Paul Kellam (University College London, UK)** summed up the meeting by saying that biologists and informaticians had 'rubbed shoulders across the disciplines' to cover a diverse and broad subject area. He praised the speakers for making the effort to place even complex and novel statistical methods into biological context. As his colleague Stewart had stressed earlier, 'Analysis means nothing unless you understand the biology'.

### References

1. Bussemaker HJ, Li H, Siggia ED. 2001. Regulatory element detection using correlation with expression. *Nature Genet* **27**(2): 167–174.
2. Collaborative Computational Project 11 website: **http://www.hgmp.mrc.ac.uk/CCP11**
3. Cuff JA, Clamp ME, Siddiqui AS, Finlay M, Barton GJ. 1998. JPred: a consensus secondary structure prediction server. *Bioinformatics* **14**(10): 892–893.
4. Golub TR, Slonim DK, Tamayo P, *et al.* 1999. Molecular classification of cancer: class discovery and class prediction by gene expression monitoring. *Science* **286**: 531–537.
5. Graz University of Technology website: **http://genome.tugraz.at**
6. HGMP website: **http://www.hgmp.mrc.ac.uk/microarray**
7. Manchester microarray website: **http://www.bioinf.man.ac.uk/microarray**
8. Stewart GR, Snewin VA, Walzl G, *et al.* 2001. Overexpression of heat-shock proteins reduces survival of *Mycobacterium tuberculosis* in the chronic phase of infection. *Nature Med* **7**: 732–737.
9. UMIST microarray website: **http://www.arrays.umist.ac.uk**

Copyright © 2002 John Wiley & Sons, Ltd. 

## References

[PDF_00313] Bussemaker H. J., Li H., Siggia E. D. (2001). Regulatory element detection using correlation with expression.. Nat Genet.

[PDF_00318] Cuff J. A., Clamp M. E., Siddiqui A. S., Finlay M., Barton G. J. (1998). JPred: a consensus secondary structure prediction server.. Bioinformatics.

[PDF_00321] Golub T. R., Slonim D. K., Tamayo P., Huard C., Gaasenbeek M., Mesirov J. P., Coller H., Loh M. L., Downing J. R., Caligiuri M. A. (1999). Molecular classification of cancer: class discovery and class prediction by gene expression monitoring.. Science.

[PDF_00329] Stewart G. R., Snewin V. A., Walzl G., Hussell T., Tormay P., O'Gaora P., Goyal M., Betts J., Brown I. N., Young D. B. (2001). Overexpression of heat-shock proteins reduces survival of Mycobacterium tuberculosis in the chronic phase of infection.. Nat Med.

